# Passivation of lanthanide surface sites in sub-10 nm NaYF_4_:Eu^3+^ nanocrystals

**DOI:** 10.1007/s11051-012-1228-3

**Published:** 2012-10-10

**Authors:** M. Banski, M. Afzaal, A. Podhorodecki, J. Misiewicz, A. L. Abdelhady, P. O’Brien

**Affiliations:** 1 Institute of Physics, Wroclaw University of Technology, Wyb. Wyspianskiego 27, 50-370 Wroclaw, Poland; 2 Center of Research Excellence in Renewable Energy, King Fahd University of Petroleum and Minerals, PO Box 1292, Dhahran, 31261 Saudi Arabia; 3 The School of Chemistry and The School of Materials, The University of Manchester, Oxford Road, Manchester, M13 9PL UK

**Keywords:** NaYF_4_, Europium, Surface site, Passivation, Nanocrystal

## Abstract

We examined in detail the optical properties of NaYF_4_:Eu^3+^ nanocrystals of ~9 nm in diameter. For such small nanocrystals roughly 17 % of Y^3+^ ions occupy surface sites and can be efficiently substituted by optically active Eu^3+^ ions. In order to determine the influence of surface Eu^3+^ on the optical properties of the whole nanocrystal, small *β*-NaYF_4_:Eu^3+^ nanocrystals with homogenous size distribution were prepared using trioctylphosphine oxide as a coordinating solvent. In order to passivate the surface sites, a thin *β*-NaYF_4_ shell was further deposited on nanocrystals core and the optical properties were investigated. For this purpose absorption, photoluminescence, photoluminescence excitation, and photoluminescence decays were recorded and analyzed. The optical characteristics of surface Eu^3+^ significantly diminish for surface passivated nanocrystals. We calculated the increase of quantum yield to the value of 64 % when NaYF_4_:Eu^3+^ core was capped by undoped shell. The optical spectroscopy techniques were shown to be sufficient in determination of surface passivation of nanocrystals with high surface to volume ratio.

## Introduction

Recently, various groups have reported the preparation of sodium yttrium fluorides (NaYF_4_) nanocrystals (NCs) in either *α* or *β* phase (Wang et al. 2010a). Yan’s group initially reported the first use of trifluoroacetate salts as precursors for one step synthesis of NaYF_4_ NCs (Mai et al. 2006). Since yttrium can be simply substituted by other lanthanide ions, the material has received considerable attention as a nanocrystalline matrix for doping by lanthanide ions. The additional advantages of the *β*-NaYF_4_ matrix arise from a wide energy bandgap (~8 eV) (Chong et al. 2007) and a very low phonon energy (~360 cm^−1^ ≈ 45 meV) (Li et al. 2007; Haase and Schäfer 2011), as compared to oxide NCs (Güdel and Pollnau 2000). Thus, the photoluminescence quantum yield (QY) in this material is high, since non-radiative relaxation of excited electrons diminishes. The reduced non-radiative relaxation rate is also responsible for the intense emission from upper excited states of lanthanide ions in a low concentration (Li et al. 2009). The same reasons also account for an efficient up-conversion emission for co-doped NaYF_4_:Yb^3+^, Er^3+^ NCs (Li et al. 2007). Nowadays, NCs of the *β* phase NaYF_4_ doped with various lanthanide ions are well-known phosphors which have found use in a variety of applications, especially as efficient markers in biological systems (Chatterjee et al. 2010).

In the search for the best capping ligands for fluoride NCs, Shan et al. proposed the use of trioctylphosphine oxide (TOPO) as a coordinating solvent in the reaction from trifluoroacetate precursors (Shan et al. 2007). Since TOPO has a higher boiling point than the previously used solvents (oleylamine, oleic acid, or octadecene) (Mai et al. 2006; Shan et al. 2007; Boyer et al. 2006), the resulting particles were more monodispersed, crystallized in the pure hexagonal phase and have found to be small enough (~10 nm) to be used as biological labels.

Small size of NCs is a requirement in bioapplications but the large number of surface atoms could have several implications. In the case of NaYF_4_, the fraction of surface Y^3+^ reaches 17 % for 8.7 nm sized NCs. For doped NCs, the percentage of doping ions on the surface can even be higher as dopants are often pushed out from the NC core (Podhorodecki et al. 2009). This leads to a decrease in QY, because of an increase in the efficiency of non-radiative relaxation by interaction of the optically active ions with surface defects as well as with organic ligands (Heer et al. 2004). Yi et al. ascribed this interaction to a significant reduction of lanthanide (Ln) emission efficiency in NCs compared to the bulk matrix (at least one order of magnitude) (Yi and Chow 2006b; Mialon et al. 2010).

In order to preserve the high PL QY, Yi et al. initially reported the synthesis of a core/shell structure composed of NaYF_4_:Yb^3+^, Er^3+^/NaYF_4_ and NaYF_4_:Yb^3+^, Tm^3+^/NaYF_4_, and showed reduced interactions of lanthanides with surface defects, ligands, and solvent (Yi and Chow 2006a). As a result, the enhancement of PL intensity increased up to 29 times when a shell was deposited on up-converting NaYF_4_:Yb^3+^, Tm^3+^ core NCs. Even bigger increase, up to 450 times, was reported by Wang et al. for shell covered small NCs (10 nm) (Wang et al. 2010b). Additionally, more complex shell compositions (e.g., NaYF_4_:Yb^3+^,Er^3+^/NaGdF_4_:Yb^3+^) were also proposed to further increase the observed PL intensity (Guo et al. 2010). However up till now, only the PL intensity was investigated in the above-mentioned studies. Moreover, the work only considered up-conversion PL and did not take advantage of crystal phase and/or oxygen-sensitive Eu^3+^ ions.

In order to correctly interpret an increase in PL intensity of colloidal NCs, its concentration has to be evaluated precisely. There is often lack of information on how the NCs concentration was calculated. To the best of our knowledge, none of the studies related to Ln doped NCs show the absorption spectra, due to difficulties associated with weak absorbing NCs:Ln^3+^. Evaluation of NCs concentration based on NCs mass is even more problematic due to differences in mass of core and core/shell NC as well as different mass contribution from surface passivated ligands. Thus, the calculation of lanthanide doped NCs concentration is a nontrivial task, leading to a significant experimental error. Therefore, the impact of the shell formation only on PL intensity can over-estimate the obtained results.

The lack of structural information brings additional uncertainty to the discussion on the origin of enhanced PL intensity. A crucial current limitation is that transmission electron microscopy (TEM) methods are generally unable to differentiate between the core and the shell structure of NaYF_4_/NaLnF_4_ NCs due to the similarity of the lattice parameters resulting in small contrast in TEM images (Abel et al. 2011; Liu et al. 2010; Cheng et al. 2010). On the other hand, techniques such as Electron Energy Loss Spectroscopy (EELS), Energy-Dispersive X-ray Spectroscopy (EDS), and X-ray photoelectron spectroscopy (XPS) provide presence and distribution of ions inside NCs. Veggel and co-workers recently carried out detailed XPS studies to determine the formation of lanthanide based core/shell particles (Abel et al. 2009). In another study, the authors have used EELS and EDS techniques and concluded that a complete and uniform shell growth does not occur on all of the NCs and an improvement in the synthesis of core/shell structure is required (Abel et al. 2011). Despite the encouraging results presented by Veggel’s group, more widely available and user-friendly techniques and protocols are required to distinguish in a routine manner the formation of shell around NC core when doped with lanthanide ions.

**Fig. 1:**
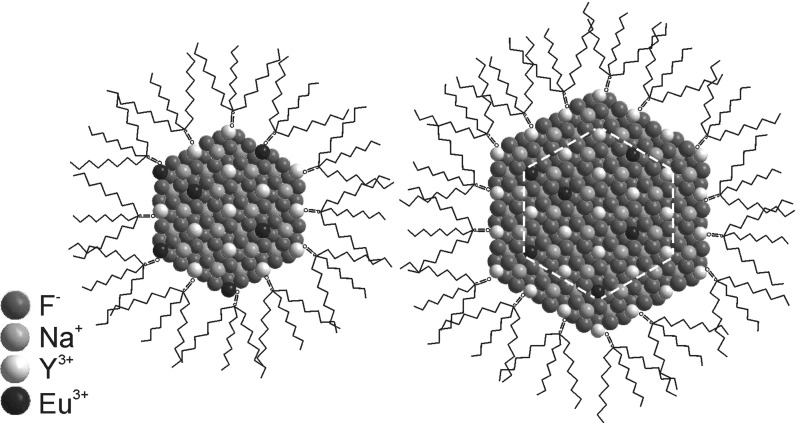
*β*-NaYF_4_:Eu^3+^ nanocrystals with europium ions on the particle surface and the same with surface passivated with undoped NaYF_4_ shell. In both cases the TOPO ligands are present on the NC surface

In this article, we have carried out detailed investigations of the optical properties of *β*-NaYF_4_:Eu^3+^ NCs and in particular Eu^3+^ ions, which are an excellent probe of a local crystal field. The europium doped NCs were passivated by NaYF_4_ shell and a broad range of optical spectroscopy experiments were conducted to distinguish between the emission properties of europium in the core and intended core/shell samples (Fig. 1). Based on the obtained results, we are able to point out the differences and propose a simple yet reliable confirmation of the surface passivation.

## Experimental section

### Synthesis of NaYF_4_:Eu^3+^ hexagonal nanocrystals

Sodium trifluoroacetate Na(CF_3_COO) (98 %), yttrium(III) trifluoroacetate hydrate Y(CF_3_COO)_3_·*x*H_2_O (99 %), europium(III) trifluoroacetate trihydrate Eu(CF_3_COO)_3_·3H_2_O (98 %), and trioctylphosphine oxide (TOPO, 90 %) were purchased from Sigma Aldrich Ltd. All chemicals were used as received. For the synthesis of *β*-NaYF_4_ nanocrystals doped with Eu^3+^ ions, a single step co-thermolysis method was utilized as described previously (Podhorodecki et al. 2012a). A mixture of 1.25 mmol Na(CF_3_COO) (0.170 g), 0.485 mmol Y(CF_3_COO)_3_ (0.207 g), and 0.039 mmol Eu(CF_3_COO)_3_ (0.022 g) was dissolved in 10 g TOPO (26 mmol) and heated up to 120 °C under vacuum for 30 min in a standard Schlenk line technique. For high quality hexagonal NCs, the growth temperature was increased to 350 °C under N_2_ within 10 min and grown for 60 min. For the precipitation of NCs, resulting solution was cooled to about 70 °C and excess dry ethanol was added followed by the centrifugation to collect the product.

### Synthesis of NaYF_4_:Eu^3+^/NaYF_4_ core/shell hexagonal nanocrystals

In order to passivated NC surface, the few monolayers of undoped NaYF_4_ were deposited on the NaYF_4_:Eu^3+^ NCs forming core/shell like structure. It was done in a two step approach. The shell precursors solution containing Na(CF_3_COO) (42.5 mg, 0.312 mmol) and Y(CF_3_COO)_3_ (51.2 mg, 0.122 mmol) dissolved in TOPO (5 g, 13 mmol) were degassed under vacuum at 120 ^°^C. After 30 min, the precursor solution was added dropwise to NaYF_4_:Eu^3+^ NCs remained at 350 °C in a 3-neck flask after the first step of synthesis. The NaYF_4_ shell was deposited on NaYF_4_:Eu^3+^ core within 30 min.

### Structural and optical characterization

Samples for TEM experiment were prepared by evaporating a dilute toluene solution of the nanoparticles onto carbon coated copper grids (S166-3, Agar Scientific), and a Philips Tecnai transmission electron microscope was used to obtain TEM images of the nanoparticles.

X-Ray powder diffraction patterns were obtained using Bruker D8 AXE diffractometer (Cu-Kα). The nanocrystalline domain size was calculated using the Scherrer equation

1 $$ T = 0.89\frac{\lambda }{{\left( {B\cos \theta } \right)}} $$

*T* is domain size to be determined, *λ* is x-ray wavelength, *B* is width of the diffraction peak in interest, and *θ* is the angle of the corresponding diffraction peak.

The PLE and PL spectra were measured using xenon lamp (450 W) coupled with a monochromator (Jobin–Yvon TRIAX 180) as an excitation source. The PL signal was recorded by CCD spectrometer (HR4000 Ocean Optics) and divided by the light source characteristic. The absorption spectra were measured on JASCO V-570 spectrophotometer. Photon Technology International Inc. systems equipped with flash xenon lamp and strobe detector both coupled with monochromators were used to observe the PL decays. PL quantum yield (PL QY) was calculated based on the experimentally determined PL decay time and calculated radiative PL decay time using the equation originally given by Verhoeven et al. (Werts et al. 2002).

## Results and discussion

### Crystal structure, shape, and size of the nanocrystals

**Fig. 2:**
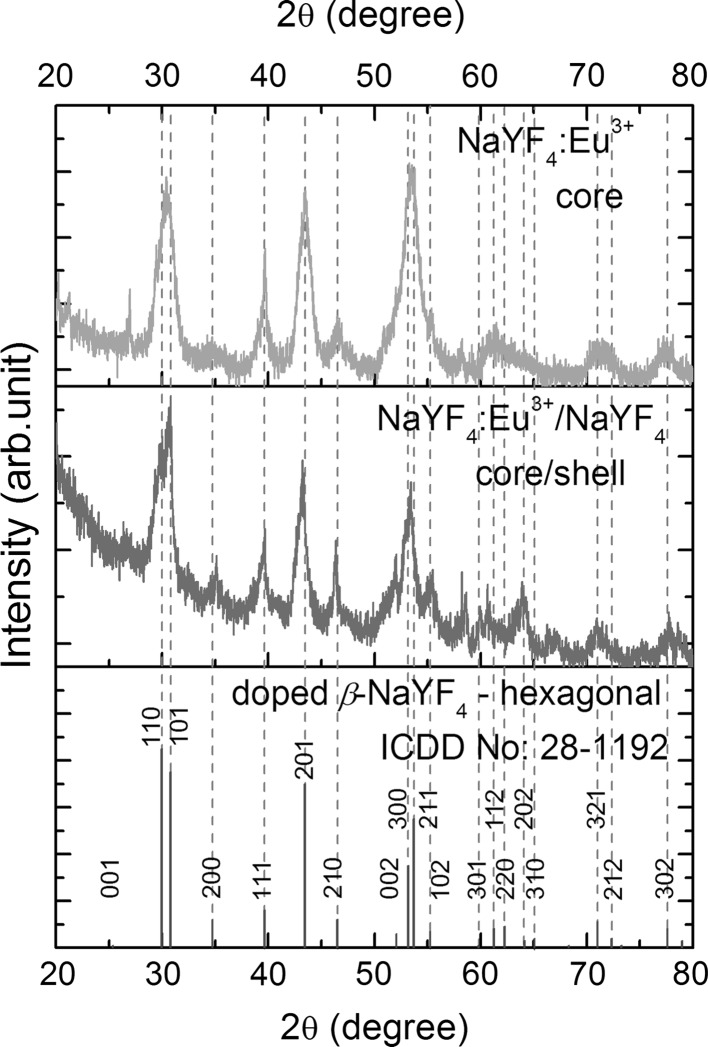
XRD spectra of core and core/shell *β*-NaYF_4_:Eu^3+^

NaYF_4_ crystals can grow in one of two crystallographic phases: the low-temperature *α*-phase (cubic) and the high-temperature *β*-phase (hexagonal) (Mai et al. 2006). However, we are particularly interested in the synthesis of hexagonal NaYF_4_ NCs because they offer significant enhancement (about 3.55 times) in PL intensity relative to the cubic phase (Yi and Chow 2006a; Wang et al. 2010b). In Fig. 2, the X-ray powder diffraction (p-XRD) data of core NaYF_4_:Eu^3+^ and core/shell NaYF_4_:Eu^3+^/NaYF_4_ samples are presented together with diffraction standard of the hexagonal NaYF_4_ lattice (ICDD No: 28-1192). The positions of recorded peaks are in good agreement with the reference pattern indicating the pure hexagonal structure of NCs. The exact positions of XRD peaks of NaYF_4_:Eu^3+^ is expected to be slightly shifted due to the Eu^3+^ ionic radius being smaller than substituted Y^3+^ ions. In our case the shifts are hardly visible, because of low europium concentration (5 %). The diffraction peaks broadening due to very small size of NCs dominates in XRD spectra. The particle sizes calculated using the Scherrer equation were 8.7 ± 0.5 and 10.2 ± 0.5 nm for core NaYF_4_:Eu^3+^ and core/shell NaYF_4_:Eu^3+^/NaYF_4_ samples, respectively. This is the evidence of increased average crystallite size for the core/shell sample.

**Fig. 3:**
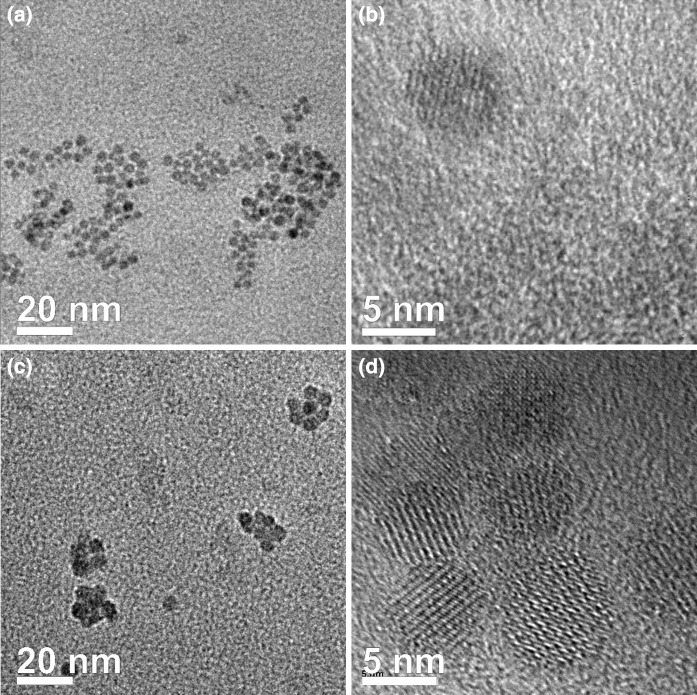
TEM images of **a**, **b** core *β*-NaYF_4_:Eu^3+^ and **c**, **d** core/shell *β*-NaYF_4_:Eu^3+^/*β*-NaYF_4_ nanocrystals

The TEM images of core and core/shell samples are shown in Fig. 3. All NCs in both samples are spherical. The TEM studies revealed that the as-synthesized hexagonal NCs are relatively monodispersed and any bimodal size distribution was not observed. Moreover, the NC diameter increases from 8.3 ± 1.7 to 10.2 ± 1.2 nm for NaYF_4_:Eu^3+^ and NaYF_4_:Eu^3+^/NaYF_4_ structures, respectively. These diameters are in-line with the XRD results. Lattice fringes corresponding to Bragg diffraction can be observed on HRTEM images (Fig. 3b, d) for both NaYF_4_:Eu^3+^ as well as NaYF_4_:Eu^3+^/NaYF_4_ NCs. This confirms the single-crystal nature of the synthesized nanostructures. Unfortunately, TEM is unable to distinguish between the core and the shell material due to similar lattice parameters (Abel et al. 2011; Liu et al. 2010). One of the goals of this study is to prove the formation of thin NaYF_4_ shell, which is responsible for passivation of surface Eu^3+^, based on careful study of optical properties.

Eu^3+^ concentration in a core NCs is expected to be 5 %, which equals the precursor EuTFA_3_ used in the synthesis. Our recent work demonstrates that the intended Eu^3+^/Y^3+^ precursors ratio corresponds well with the final composition of Eu^3+^ doped NaYF_4_ NCs (Podhorodecki et al. 2012b). The level of doping was chosen to observe intense emission from the ^5^D_0_ and ^5^D_1_ levels.(Chong et al. 2007; Podhorodecki et al. 2012b).

*β*-NaYF_4_ crystallizes in a hexagonal structure in which Eu^3+^ ions can occupy three sites. The two main sites are of *C*_*3h*_ symmetry, which should induce a strong asymmetric splitting of the ^7^F_J_ multiplet due to axial character of their symmetry. It is also possible that Eu^3+^ substitutes Na^+^ at the *2h* site with *C*_*3*_ symmetry. However, this situation is less probable, especially in a matrix with low doping percentage, and will not be considered in the present work. (Zakaria et al. 1997). Thus, if Eu^3+^ occupy any other sites than *C*_*3h*_, they can be related to ions localized on the NCs surface.

## Optical properties

**Fig. 4:**
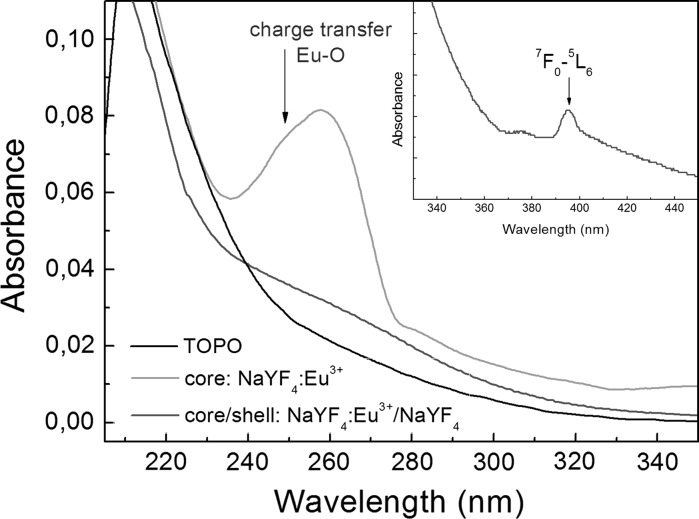
The absorption spectra of core, core/shell NCs, and TOPO molecules

The optical properties were investigated to examine the influence of Eu^3+^ located on the surface on the excitation and emission of NCs smaller than 10 nm in diameter. We started our investigations with the absorption spectra measurements which are often ignored in the lanthanide doped fluoride NCs. The NaYF_4_ crystals exhibit a large energy gap (~8 eV), thus absorption edge is not expected in UV–Vis range. Moreover, due to the very small value of Ln absorption cross section, the absorption bands related to *f*–*f* transitions are strongly limited. (Carlos et al. 2009). However, we were able to observe the ^7^F_0_–^5^L_6_ transition for the high concentrations of NaYF_4_:Eu^3+^/NaYF_4_ NCs (inset of Fig. 4) which confirms a relaxation of selection rules making *f*–*f* transitions slightly probable when Eu^3+^ occupy non-centrosymmetric crystal sites. In the inset of Fig. 4, we can notice that the band of ^7^F_0_–^5^L_6_ transition is settled on another wide absorption band. In order to determine its origin, the absorption spectrum was recorded in a wider spectral range. Fig. 4 shows the absorption spectra of core and core/shell NCs capped by TOPO and TOPO itself. It is clearly visible that the absorption of core/shell NCs is determined by absorption of TOPO ligands. The spectrum of core only has additional absorption band at around 250 nm. This band is significantly wider and much more intensive in comparison with *f*–*f* transitions bands. We attribute this band to a transition between the ground and the charge-transfer state of the Eu–O bond (CT). We presented the detailed discussion about parameters influencing the exact CT position in our previous work (Banski et al. 2010). Moreover, the observed position of absorption band is in a good agreement with the literature values equal to 240–255 nm for Y_2_O_3_:Eu^3+^ (Shang et al. 2011).

On the XRD spectra there were no diffraction peaks corresponding to residual Ln_2_O_3_ phase. Moreover, the CT band totally disappeared for surface passivated sample, what suggests that all Eu–O bonds are efficiently broken. It confirms that the CT band is due to interaction of Eu^3+^ on the NCs surface and oxygen from the P=O group of TOPO surface ligands. The absorption spectra can be used in a simple way to prove the passivation of surface sites in Eu^3+^ doped fluoride NCs.

The photoluminescence excitation (PLE) experiment is much more sensitive to the optical properties for weakly absorbing materials. Hence, the PLE spectra corresponding to the direct excitation of the Eu^3+^ ions in NaYF_4_:Eu^3+^ and NaYF_4_:Eu^3+^/NaYF_4_ NCs were recorded.

**Fig. 5:**
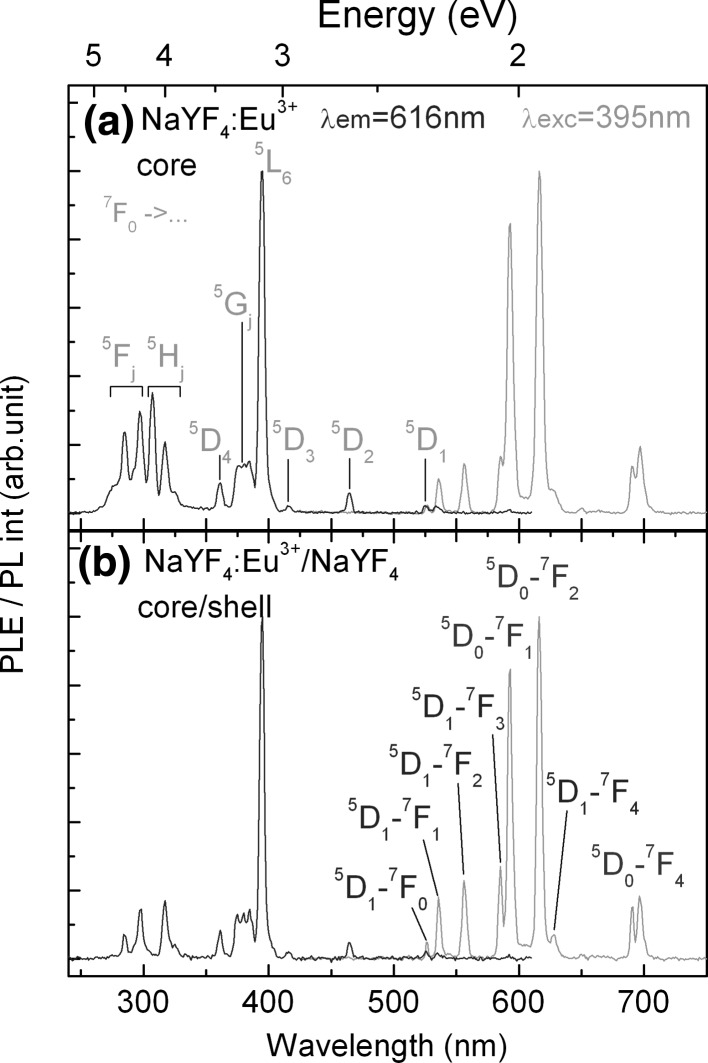
PLE and PL spectra of **a** *β*-NaYF_4_:Eu^3+^ and **b** *β*-NaYF_4_:Eu^3+^/*β*-NaYF_4_ NCs

The PLE spectra presented in Fig. 5 (PL intensity monitored at 616 nm) clearly show that direct ^7^F_0_–^5^L_6_ transition at 395 nm wavelength causes the most intensive Eu^3+^ excitation in both samples. Other excitation bands associated with *f*–*f* transitions are also present. At longer wavelengths, the transitions from ^7^F_0_ to ^5^D_1_, ^5^D_2_ and ^5^D_3_ states are present at 527, 464 and 416 nm, respectively. At higher energy, the excitations through the following transitions ^7^F_0_–^5^G_j_, ^7^F_0_–^5^D_4_, ^7^F_0_–^5^H_j_, and ^7^F_0_–^5^F_j_ are possible at 373–387, 360 and 275–325 nm, respectively (Gao et al. 2009). However, all of these excitation bands are ~3–20 times less intensive. The full width at half maximum (FWHM) of ^7^F_0_–^5^L_6_ in *β*-NaYF_4_:Eu^3+^ NCs is only 4.7 nm, which reduces to 3.8 nm after the shell formation. The small FWHM confirms the high quality of nanocrystal structure.

The PLE spectra of *β*-NaYF_4_:Eu^3+^ and *β*-NaYF_4_:Eu^3+^/*β*-NaYF_4_ samples are quite similar. Due to the low symmetry of the crystal field of *β*-NaYF_4_:Eu^3+^ NCs, the ^5^H_J_ and ^5^F_J_ terms are split into separate energy levels: 285, 297, 307 and 317 nm. After the shell formation, the excitation band at 307 nm disappears, which can be associated with the reduction of Eu^3+^ on the surface site.

In the PL spectra given in Fig. 5, many well-resolved emission bands corresponding to the electronic and magnetic dipole transitions from ^5^D_1_ and ^5^D_0_ states to ^7^F_j_ manifolds can be distinguished. The PL peak positions of all the observed bands are consistent for both samples. The green emission from upper excited state (^5^D_1_) is possible due to the limited phonon assisted relaxation rate in the high quality *β*-NaYF_4_ matrix. It indicates that the emission from ^5^D_1_ and ^5^D_0_ states is competitive.

The surface passivation causes an increase in the intensity ratio of the electric dipole transitions from the lowest and upper excited state (^5^D_1_–^7^F_3_/^5^D_0_–^7^F_2_). Two possible phenomena could cause this observation. First, when *β*-NaYF_4_ shell was formed, some of Eu^3+^ ions may diffuse from the core to the shell (Dong and van Veggel 2008). The separation between the ions increases and the probability of cross-relaxation processes diminishes. As only a thin layer of shell is applied (~1.5 nm from XRD, ~1.9 nm form TEM), the diffused Eu^3+^ ions would, in fact, again occupy the surface sites. Hence another mechanism takes place in this case. Excited Eu^3+^ ions located on the surface sites interact with higher energy surface phonons and/or ligand molecules, and the nonradiative relaxation from ^5^D_1_ state is more prominent due to multi-phonon transitions. A thin *β*-NaYF_4_ shell successfully passivates the surface active sites and separates Eu^3+^ ions from TOPO and solvent. As a result, the green emission from ^5^D_1_ of Eu^3+^ state of core/shell NCs increased, which is an important evidence for the shell formation. Moreover, this explanation is consistent with conclusions derived from absorption measurements.

The characteristic FWHM parameters of the magnetic and electric dipole transitions presented in the PL spectra (Fig. 5) are reduced by 1 nm when the shell is formed (5.86–4.87 nm and 5.63–4.65 nm for ^5^D_0_–^7^F_1_ and ^5^D_0_–^7^F_2_ transitions, respectively). This observation is yet another indication that the number of Eu^3+^ occupied sites was reduced by the surface passivation.

**Fig. 6:**
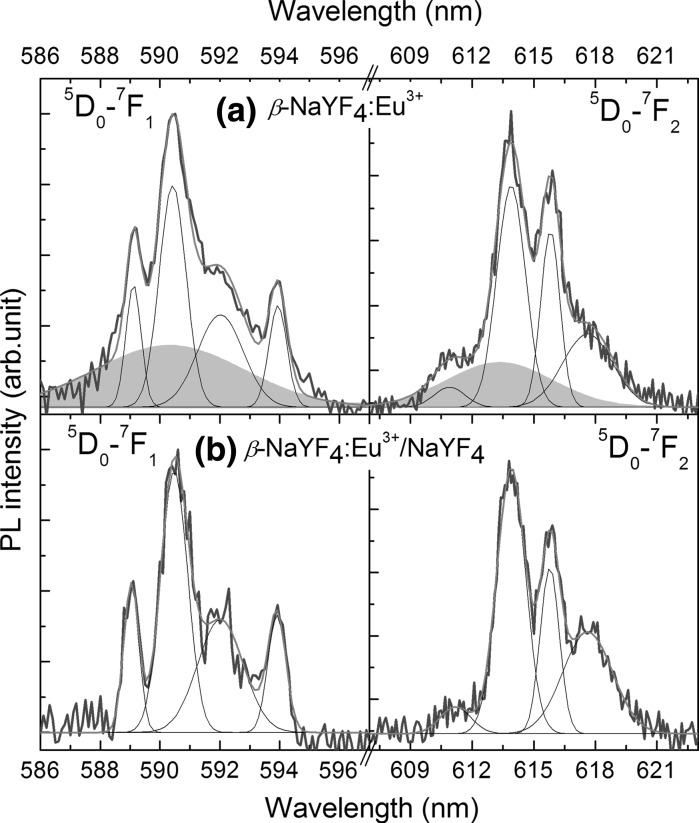
High resolution PL spectra of ^5^D_0_–^7^F_1_ and ^5^D_0_–^7^F_2_ transitions of **a** core and **b** core/shell *β*-NaYF_4_:Eu^3+^. The emission bands related to the Eu^3+^ on the NC surface are shaded

In order to closely look at the emission properties, the PL spectra of ^5^D_0_–^7^F_1_ and ^5^D_0_–^7^F_2_ transitions were recorded with 0.3 nm resolution (Fig. 6). Crystal field theory predicts that for ^5^D_0_–^7^F_2_ transition of Eu^3+^ in a *C*_*3h*_ site, we should observed only one emission peak (Ju et al. 2009). However, for the nanocrystalline matrix the symmetry of Eu^3+^ sites could be reduced to *C*_*3*_ or even *C*_*S*_ symmetry with the number of observed PL lines increasing to five (Ju et al. 2009). The PL spectra of both samples are presented in Fig. 6. They are composed of four emission bands, and they look similar, at a first glance. However, the difference between *β*-NaYF_4_:Eu^3+^ and *β*-NaYF_4_:Eu^3+^/*β*-NaYF_4_ NCs is well-resolved. The ^5^D_0_–^7^F_1_ as well as the ^5^D_0_–^7^F_2_ transition in the core sample contains wide emission band cantered at 590.3 and 613.3 nm, respectively. These bands diminished in the core shell/sample. We ascribed their origin to Eu^3+^ ions on the NC surface, which become passivated by *β*-NaYF_4_ shell. The other narrow PL peaks did not change after shell formation, so they can be related to the well-defined internal sites of *β*-NaYF_4_ matrix.

**Fig. 7:**
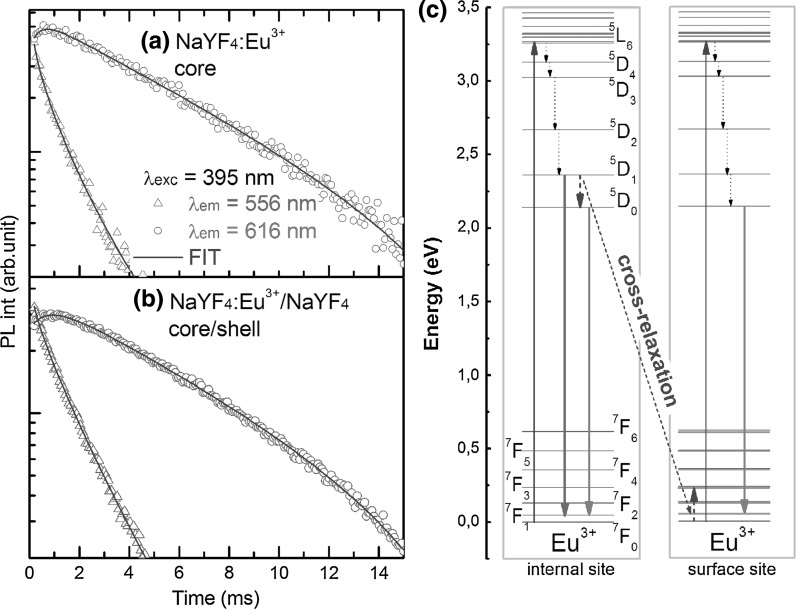
PL decay of core (*β*-NaYF_4_:Eu^3+^) **a** and core/shell (*β*-NaYF_4_:Eu^3+^/*β*-NaYF_4_) **b** NCs. Energy levels diagram with radiative and nonradiative transition in Eu^3+^ on internal and surface sites **c**

Complementary results were also obtained from PL decay experiments (Fig. 7). The PL decay time of the upper ^5^D_1_ state is driven by cross-relaxation between ions and phonon related to non-radiative processes. Considering that shell formation influences the relaxation related to ions interactions with surface phonons and/or ligand molecules, an elongation of PL lifetime is expected. We fitted PL decays of ^5^D_1_ state with stretched exponential functions.

2 $$ I_{PL} \left( t \right) = I_{0} \frac{\beta }{\tau }\left( {\frac{t}{\tau }} \right)^{\beta - 1} \exp \left( { - \left( {\frac{t}{\tau }} \right)^{\beta } } \right)^{{}} $$

where *τ* is the PL lifetime, *β* can be interpreted as a disorder parameter, and *I*_0_ is a constant. A slight elongation of the PL decay time from 1,325 to 1,623 μs was observed for core and core/shell samples, respectively (Table 1). Moreover, the *β* parameter, which estimates the uniformity of Eu^3+^ ions environment in NCs increased from 0.835 to 0.912 after surface passivation. This further confirms our expectation according to homogeneous shell formation during surface passivation processes. The elongation of decay and rise times of PL from ^5^D_0_ state are other characteristic observations.

**Table 1:** PL decay times (τ), disorder parameters (β), and QYs of NaYF_4_:Eu^3+^ and NaYF_4_:Eu^3+^/NaYF_4_ samples

	^5^D_1_–^7^F_2_ (556 nm)		^5^D_0_–^7^F_2_ (616 nm)		QY (%)
	τ_1_ (μs)	*β*	τ_1_ (μs)	τ_2_ (μs)	
NaYF_4_:Eu^3+^ (5 %)	1,325 ± 73	0.835 ± 0.014	−434 ± 62	6,170 ± 170	56.8
NaYF_4_:Eu^3+^ (5 %)/NaYF_4_	1,623 ± 32	0.912 ± 0.005	−828 ± 52	7,288 ± 150	64.0
NaYF_4_:Eu^3+^ (2 %)	2,686 ± 38	0.828 ± 0.025	−1,017 ± 120	7,782 ± 140	

Recently, the cation exchange was suggested to form a gradient alloy structure, when the core/shell fluoride nanocrystals were expected (Dong et al. 2011). The authors concluded that this mechanism can significantly deplete the Eu^3+^ concentration in a NC core when undoped shell is intended to be deposited. As a result, any changes in the optical properties due to a reduction of the ion–ion interaction are incorrectly interpreted as a proof of shell formation.

In order to exclude the cation exchange as a possible mechanism for improvement in the optical properties of our core/shell structure, we evoke our results of PL decay times of NaYF_4_ NCs doped with 2 % of Eu^3+^. The doping level was chosen to be 2.5 times lower than the original concentration, because the volume of core/shell NCs increases two times compared to core ones. For 2 % sample, in a comparison with 5 % core sample, the τ_PL_ decay time will increase due to bigger inter ionic distances, which are supposed to reduce the efficiency of Eu^3+^–Eu^3+^ cross relaxation. The determined value of PL decay times (τ_PL_) are 7,782 and 2,682 μs for emission from ^5^D_0_ and ^5^D_1_, respectively. Both of them significantly exceed the values obtained for core as well core/shell sample (Table 1). These results indicate that during shell formation the cation diffuse was not sufficient to separate Eu^3+^ ions efficiently, thus the observed changes of PL spectra have to arise directly from surface passiation.

Finally, the photoluminescence quantum yield (PL QY), defined as QY = *τ*_*PL*_/*τ*_*R*_ *100 *%,* was calculated to assess the improvement in optical properties of NaYF_4_:Eu^3+^ NCs after the shell formation. The modified equation (eq. 2) originally given by Verhoeven et al. was used to calculate radiative decay time from ^5^D_0_ state (*τ*_R_) (Werts et al. 2002).

3 $$ \tau_{R} = \frac{1}{{A_{MD,0} \cdot \varepsilon_{\text{solvent}}^{3/2} }} \cdot \left( {\frac{{I_{MD} }}{{I_{TOT} }}} \right) \cdot \left[ {\frac{{\varepsilon_{\text{matrix}} + 2\varepsilon_{\text{solvent}} }}{{3\varepsilon_{\text{solvent}} }}} \right]^{2} $$

*ε* is the dielectric constant of NaYF_4_ matrix and cyclohexane equal 2.477 and 2.023, respectively (Lage et al. 2005), *A*_MD,0_ is the spontaneous emission probability for ^5^D_0_–^7^F_1_ transition in a vacuum (14.65 s^−1^), and (I_TOT_/I_MD_) is the ratio of the total Eu^3+^ emission spectrum to the area of the ^5^D_0_–^7^F_1_ band (Vela et al. 2008). The calculated values of radiative lifetime are *τ*_*R*_ = 10.87 ms (NaYF_4_:Eu^3+^) and *τ*_*R*_ = 11.39 ms (NaYF_4_:Eu^3+^NaYF_4_). Taking values of *τ*_*PL*_ from PL decay experiments, the PL QY was calculated to be 57 % for the core and 64 % for the core/shell. This observation implies that the shell formation results in an increase in the efficiency of Eu^3+^ luminescence.

## Conclusions

In our investigations of Eu^3+^ doped NaYF_4_ NCs, we observe a significant absorption band in the UV range. We attribute this to the Eu–O charge-transfer state, which arises from the interaction between oxygen (from trioctylphosphine oxide) and Eu^3+^ at the nanocrystal surface. For NCs smaller than 10 nm, due to their high surface to volume ratio, charge transfer becomes an important absorption mechanism. We have shown that the surface of NaYF_4_:Eu^3+^ NCs can be successfully passivated by a thin layer of undoped matrix. This was confirmed by the absence of CT band in the absorption spectra. Moreover, the surface passivation modifies crystal field influence on the significant fraction of Eu^3+^ ions. This was observed as changes in PL and PLE characteristic features, ED/MD ratio and peaks broadenings, which are discussed in details. The change in the emission lifetime is determined as well, which is used to calculate an increase in the PL emission efficiency up to a value of 64 % for surface passivated NaYF_4_:Eu^3+^/NaYF_4_ NCs.
